# Effect of combined training in water on hippocampal neuronal Plasticity and memory function in healthy elderly rats

**DOI:** 10.3934/Neuroscience.2024017

**Published:** 2024-08-21

**Authors:** Roya. Askari, Mohadeseh. NasrAbadi, Amir Hossein. Haghighi, Mohammad Jahan Mahin, Rajabi Somayeh, Matteo. Pusceddu

**Affiliations:** 1 Department of Exercise Physiology, Faculty of Sport Science, Hakim Sabzevari University, Sabzevar, Iran; 2 Department of Exercise Physiology, Faculty of Human Sciences, Shahrood Branch, Islamic Azad University, Shahrood, Iran; 3 The Sports Physiology Laboratory, University of Cagliari, Italy

**Keywords:** combined water-based training, CREB, NGF, memory, aging

## Abstract

**Purpose:**

The cyclic AMP response element–binding protein (CREB) and nerve growth factor (NGF) have been proposed as key modulators of brain health and are involved in synaptic plasticity. The study investigates how combined water-based training affects hippocampal neuron plasticity and memory function in old rats.

**Methods:**

16 Wistar male rats 24-month-old were randomly divided into two groups: combined training (n = 8) and control (n = 8). Four sessions were performed per week for 10 weeks, and consisted of resistance and endurance training in water. The control group was placed in a water container during training for 30 minutes to be homogenized in terms of the stress conditions. The.NGF and CREB genes in the hippocampus were evaluated and the working memory was measured using real-time PCR and Y-maze tests. The SPSS 26 software was utilized in which independent t-tests were used to analyze the genes and the Mann-Whitney U test was used to analyze functional memory with a significant level of (P < 0.05).

**Results:**

The combined training resulted in a significant rise in NGF and CREB gene expression in the hippocampus tissue of elderly rats compared to the control group (P < 0.05); however, there was no notable difference in the Y maze performance test between the two groups (P < 0.05).

**Conclusions:**

These findings suggest that water-based combined training has beneficial effects on gene expression of NGF and CREB; however, it is necessary to conduct more studies to comprehend the effects of combined training on memory function.

## 1. Introduction

According to the World Health Organization, healthy aging is defined as “the process of developing and maintaining functional ability that promotes well-being in the elderly” [1]. Aging causes physiological changes in various systems of the body, especially the nervous system. The set of changes that occur in the brain with age reduces cell-to-cell efficiency (cellular communication), and thus the ability to remember and learn [2]. Although inactivity can lead to decreased physical fitness and the incidence of chronic diseases in older adults [3], regular physical training leads to increased functional activity, improved health, and optimal social relationships, and thus, the opportunity to experience healthy aging [1]. Physical activity enhances [4] and thereby counteracts an age-related cognitive decline [5]. The beneficial effects of exercise activity on brain structure and cognitive function occur through improved levels of neurotrophins in the brain [6].

Neurotrophins, such as brain-derived neurotrophic factor (BDNF) and neural growth factor (NGF), have been proposed as key brain health regulators and are involved in neurogenesis, neuronal survival, and synaptic plasticity [5],[7]. NGF is important in neuronal health [8],[9]. Reduced NGF levels have been observed in the hippocampus of older mice and may be implicated in age-related cognitive function [8]. Additionally, NGF helps in the survival and regeneration of neurons during aging and in age-related diseases such as Alzheimer's [10]. Several studies have reported an increase in the NGF protein within the blood and brain after various exercise activities in humans and animals [11]–[14].

The cyclic AMP response element–binding protein (CREB) is a cellular transcription factor that binds to a specific sequences of DNA, called cyclic adenosine monophosphate-responsive elements (cAMP), and either increases or decreases the transcription of upstream or downstream genes [15]. Increased calcium and cAMP concentrations in the hippocampus can lead to phosphorylation and CREB activation. This transcription factor is a component of intracellular signaling events that regulate various biological processes, including memory, and plays an undeniable role in neuroplasticity, long-term memory formation in the brain, and spatial memory [16]. Some genes regulated by CREB include BDNF and NGF. Various studies have shown that different types of exercises have increased CREB expression and improved the memory and cognition of elderly mice [17]–[21].

Currently, training in water is considered a safe and useful alternative to dry training because of the nature of weight intolerance. Because of the high density and viscosity of water, the forces exerted on one's joints are less; therefore, these types of trainings are beneficial for the elderly population [22]. However, studies that evaluated the effects of water training in the elderly are very limited. For instance, training in water improves attention and memory [23]. Ultimately, CREB and BDNF improve hippocampus-dependent memory in older mice [20].

Although the relationship between training and memory has been proven, the pathways involved in this connection and the intensity, the duration, and the type of optimal training remain unknown. Because water training is safe for the elderly, and a combination of resistance and endurance training in water can simultaneously benefit the elderly from the effects of adapting to both resistance and endurance training [24], the role of combined training (resistance and endurance), especially in the elderly, can be a good strategy to achieve these beneficial effects. This study aims to investigate the effects of 10 weeks of combined water training on hippocampal neural plasticity (CREB and NGF) and memory function in elderly rats.

## 2. Materials and methods

### 2.1. Samples

This was an experimental study with a post-test design and a control group. Sixteen 24-month-old male Wistar rats that weighed 315–325 gr were selected and randomly divided into two groups: combined training in water (n = 8) and control (n = 8). The rats were placed at a temperature of 30 ± 1 °C, humidity of approximately 45%, and a cycle of 12 to 12 h; the cages were made of Plexiglas with a net door of dimensions 43 × 27 × 25 cm and provided access to standard food and water. The animals from the training group performed physical training in water with temperatures at 30 °C in a glass container of 100 cm long, 50 cm high, and 50 cm wide [25]. The water depth was adjusted based on the rat body length. No regular physical activity was observed in the control group. For homogenization, during exercise training (i.e., for 30-minutes), they were placed in a water container at a depth of five centimeter.

The study was approved by the research ethics committee of Sabzevar University of Medical Sciences (IR. HSU. AEC.1401.004).

### 2.2. Combined training protocol in water

**Figure 1.**
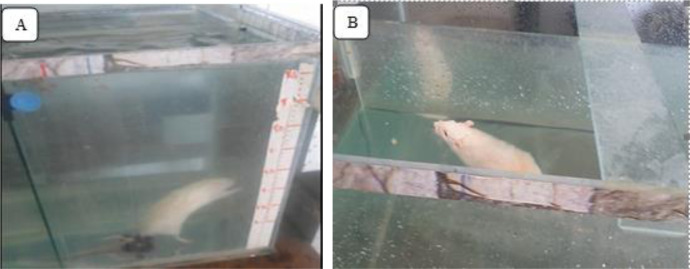
A) Resistance training section, B) Endurance training section.

The rats alternately performed a combination of resistance and endurance training programs for four days a week (every other day). A mesh metal grating (a distance of 2 cm from each grid similar to the ladder of rat special resistance training) [24] was attached as a ladder to the container wall (Figure 1A). During resistance training in the first week, the animals performed five sessions of familiarity with the water environment and climbing the ladder (Table 1). In the first meeting of the week, the rats' release distance was close to the ladder so as to climb it with the least immersion; in the following sessions, this distance increased. After the familiarization period, the water level was adjusted to approximately 200% of the animal's body length, and the rats were released from a distance of 35 cm to climb the ladder. The selection of this interval was based on the duration of hypoxia under water so as to not exceed 10 seconds over 10 weeks. Weights were attached to the tail of rats by a band; before applying the new training load after two weeks, the weight of the animal was measured on a laboratory scale, and the weight was determined based on the new weight [24].

Similar to resistance training, endurance training was followed by five days a week of familiarity, as shown in Table 1 [26]. After familiarization with the endurance training, the main program was implemented in water with a height equal to 140% of the rat's body length [27]. During endurance training, the glass container was divided into two parts (dimensions 25 × 100 cm), and each rat swam in a separate line (Figure 1B).

**Table 1.** Combined training program (resistance-endurance) in water.

	**Familiarization course of resistance training in water (one week)**	**Familiarization course of endurance training in water (one week)**
**Session 1**	Climbing the ladder with a weight equal to 10% of the rat's body weight, 3 sets with 8 repetitions and 1 Minute rest between each set with a water height of 100% of the rat's length.	Swimming for 10 minutes with a water height of 100% of the rat's body length
**Session 2 & 3**	Climbing the ladder with a weight equal to 15% of the rat's body weight, 3 sets with 8 repetitions and 1 Minute rest between each set with a water height of 120% of the rat's length.	Swimming for 15 minutes with a water height of 120% of the rat's body length
**Session 4 & 5**	Climbing the ladder with a weight equal to 20% of the rat's body weight, 3 sets with 8 repetitions and 1 Minute rest between each set with a water height of 140% of the rat's length.	Swimming for 20 minutes with a water height of 140% of the rat's body length

	**Resistance training Program**				**Endurance training Program**

**Weeks**	**Set**	**Repetition**	**Rest between each set**	**Amount of weight based on body weight**	**Duration of continuous swimming**
**Week 1 & 2**	4	10	1 Minute	30%	30 Minute
**Week 3 & 4**	4	10	1 Minute	35%	35 Minute
**Week 5 & 6**	4	10	1 Minute	40%	40 Minute
**Week 7 & 8**	4	10	1 Minute	45%	45 Minute
**Week 9 & 10**	4	10	1 Minute	50%	50 Minute

### 2.3. Control Group

The control group was placed in a 5 cm deep water container for 30 min in order not to differ from the training group in terms of the stress conditions [28].

### 2.4. Working Memory test

**Figure 2.**
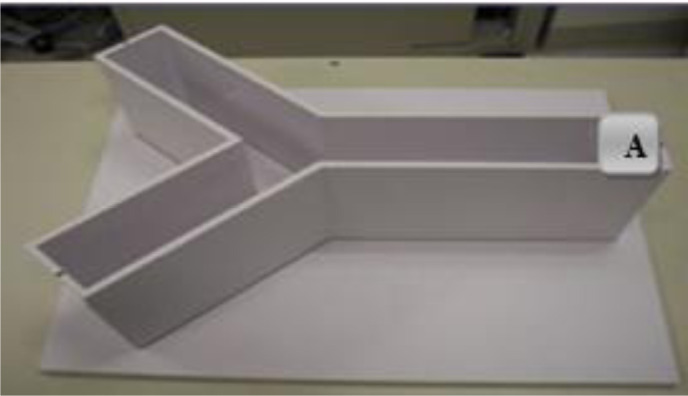
Y-Maze.

The working memory was measured using the Y-maze test and alternation percentages. The working memory was assessed by observing and measuring the spontaneous alternation behavior during a working session. The maze was made of Plexiglas; each arm was 15 × 30 × 40, and the arms were connected through a central area. Each rat was placed at the end of one arm of maze (A) and allowed to move freely in the arms for 8 min (Figure 2). The number of animals that entered each arm was recorded. An alternate behavior was considered successful, and a serial entry was added into all arms in the triple sets. Thus, the alternation percentage that determined the spatial memory in the animal according to formula 1 was the number of non-repetitive triad arms divided by the total arms of the triads minus 2; because it was expressed as a percentage, the total data was multiplied by 100 [29].

Formula 1: Spatial memory percentage = number of non-repetitive triad arms/ (total number of triad arms – 2) × 100.

### 2.5. Tissue sampling and gene expression measurement

Forty-eight hours after the last training session and 10 to 12 h of fasting, the animals were anesthetized with chloroform, the animal heads were removed, and the hippocampal tissue was separated. The samples were stored at −80 °C, and gene expression was analyzed by Real-Time PCR. First, all the necessary ingredients for the PCR were removed from the freezer, vortexed (Kiagene Co., Iran), spun, and stored on ice. Then, for each gene, a mixture of different PCR components was prepared; after mixing and spinning, 9 µl was distributed into the microtubes of the device, and in each vial, a microliter of cDNA sample was added (the final volume of each PCR reaction was 10 µl).

To extract RNA from the tissues and to evaluate gene expression by Real-Time Polymerase Chain Reaction (RT-PCR), biological samples were prepared beforehand. The cell surface area was depleted. The cells were washed with PBS buffer, and after discharging the PBS, a certain amount of triazole (Kiazist Co., Iran) (1 mM of triazole in a container of 10 cm) was poured onto the cells to lyse them. The cell lysate was collected in a 1.5 ml microtube. For non-adhesive eukaryotic and bacterial cells, the existing cell suspension was centrifuged (Hettich Co., Germany), the cell surface was discarded, and the cell sediment was lysed in a certain amount of triazole (1 mM triazole per 5–10 million cells). For bacterial cells, one mM of triazole was added per 10 million cells. The resulting cell lysate was collected in a 1.5 ml microtube.

For converting RNA to cDNA, all the Easy cDNA Synthesis Kit materials (Kiatous, Iran) were removed from −20 °C and the RNA samples were removed from −70 °C and transferred to ice after melting. All materials were shortened and spun before overtaxing. To prepare the reverse transcriptase (RT) mixture, cDNA materials including RT buffer, RT enzyme, Oligo dT primer, and diethyl pyro carbonate (DEPC)-treated water were mixed and then distributed in 9 µl volumes into 0.2 ml microtubes. Prepared microtubes that contained RT mix and the RNA samples were placed in a real-time PCR thermocycler (ABI Stepone Co., USA), and temperature change programs were implemented. The cDNA samples were stored at −20 °C. The sequences of the primers and probes used are presented in Table 2.

**Table 2.** The sequence of the investigated primers and probes.

Host	Gene	Primer	Oligo	Length
Rat	CREB	Forward Primer	AAGCAGTGACGGAGGAGCTT	20
		Reverse Primer	CATGGATACCTGGGCTAATGTGG	23
Rat	GAPDH	Forward Primer	AGGTCGGTGTGAACGGATTTG	21
		Reverse Primer	TGTAGACCATGTAGTTGAGGTCA	23
Rat	NGF	Forward Primer	ACAGGCAGAACCGTACACAG	20
		Reverse Primer	CTATTGGTTCAGCAGGGGCA	20

After the experiment, the data, including the threshold cycle (CT) and replication and melting curves of each gene, were prepared for analysis. The CT numbers of the reference gene and the main gene of each sample were determined, and the relative expression of each gene was calculated using the ^2−ΔΔ^ CT formula to examine the fold-change of each gene.

### 2.6. Statistical Method

Descriptive and inferential statistics were used to analyze the data. The Schapiro-Wilk test was used to normalize the data. In the case of normality independence, a t-test was used; otherwise, the Mann-Whitney U test was used. The Leven test was used to determine the homogeneity of variance. Data were analyzed using the SPSS 22 software at a significance level of P < 0.05.

## 3. Results

Table 3 presents the mean and standard deviation of the hippocampal tissue variables, including NGF, CREB, and working memory.

**Table 3.** Mean values and results of Independent T Test for NGF, CREB gene expression. Mann-Whitney U Test in working memory of two groups.

**Independent T test**						
Variable		Groups	N	Mean ± std. Deviation	t	**P**
**NGF** Fold change		Control Training	8	1/01 ± 0/15	-4/09	0/001*
			8	1/32 ± 0/15		
**CREB** Fold change		Control Training	8	1/16 ± 0/48	-2/53	0/024*
			8	3/14 ± 2/16		

**Mann-Whitney U test**						

Variable	group	N	Mean ± std. Deviation	Mean Rank	Z	**P**
**Working memory** (%)	control training	8	42/4 ± 60/56	6/40	-0/453	**0/651**
		8	44/4 ± 25/90	7/38		

* Significance at P < 0.05 level

A parametric independent t-test was used to analyze NGF and CREB gene expression, and the non-parametric Mann-Whitney U test was used to compare the means for working memory.

**Figure 3.**
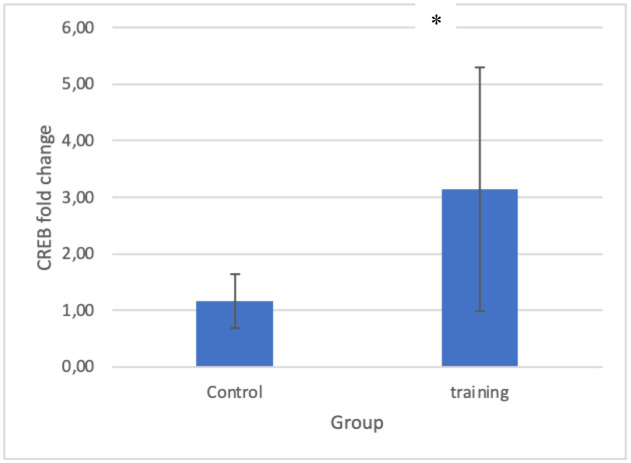
fold change in expression of CREB.

**Figure 4.**
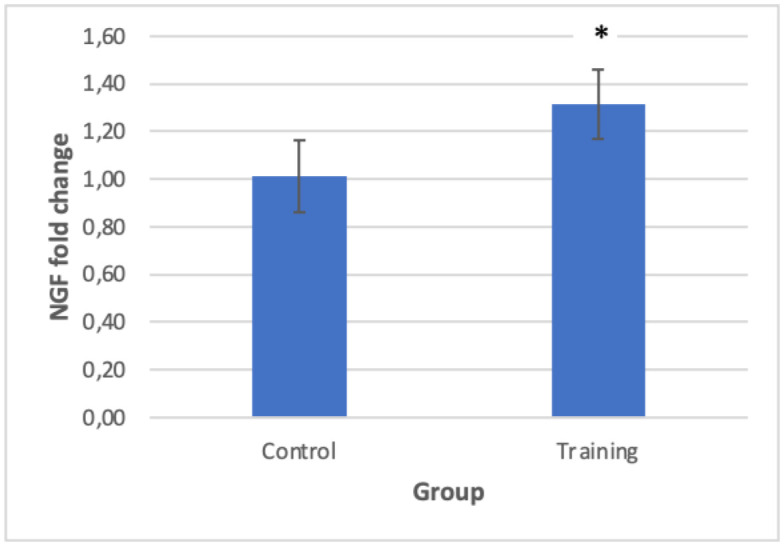
fold change in expression of NGF.

NGF gene expression was significantly higher in the training group than in the control group (P = 0.01) (Figure 3 and Table 3). In addition, CREB gene expression in the training group showed a significant increase in the training group compared to the control group (P = 0.024) (Figure 4), which indicates that 10 weeks of combined training increased NGF and CREB gene expression in the hippocampus of elderly rats.

Although the combined training in water increased memory performance, this increase was not statistically significant in the working memory test (P > 0.05) (Table 3).

## 4. Discussion

### 4.1. CREB and NGF expression

Several studies have demonstrated that regular exercise may affect neuronal function in older adults [18],[20],[30],[31].

In investigating the relationship between NGF and CREB and their possible activation mechanisms, the activation of the NGF receptor tropomyosin receptor kinase A (TrkA) stimulates three main signaling pathways—Mitogen‑activated Protein kinase (MAPK), PLC-γ, and AKT—which leads to the initiation of a cascade of intracellular events characterized by changes in the expression of the genes responsible for survival, growth, and differentiation. In the MAPK pathway, phosphorylated RSK is transferred to the nucleus for CREB phosphorylation. The CREB transcription factor hereby participates in protein translation and the control of gene transcription [32],[33]. Therefore, in the present study, the increased expression of NGF observed with the combined swimming training may lead to the positive regulation of the expression of downstream proteins such as CREB, and consequently, nerve cell survival and synaptic plasticity.

Our findings showed that the combined training in water had a significant effect on hippocampal CREB gene expression in elderly rats. In line with our results, four weeks of swimming training (6 days Per week for 60 min) during the aging process increased the protein levels and the mRNA expression of CREB [18]. In another study, eight weeks of training in water (five days per week for 30 minutes a day) led to increased CREB, BDNF, and the activation of Sirt-1 signaling pathways in the hippocampus [20]. Additionally, in another study, the CREB levels increased after both aerobic and resistance training protocols [21]. Contrary to our data, high-intensity aerobic training did not negatively affect the CREB pathway or significantly alter memory function in mice [34]. It should be noted that the evaluation of the above research was conducted in the striatum, which is different from our evaluation of the hippocampal tissue. The reason Aguiar et al.'s (2010) results contradict the current study may be due to the type (sprint interval running on a treadmill) and intensity of the training. Another study conducted by the same group (Aguiar 2011) showed that lower intensity aerobic training in older rats improved spatial memory through increased hippocampal CREB [17]. We suggest that training in water or moderate-intensity training has better effects than strenuous training, and that the nature of exercise is effective.

Exercise training is thought to activate neurogenesis through various pathways [35]. One of the important signaling pathways in the brain proposed over the years is the binding of BDNF to its specific receptor in different regions of the hippocampus [36]. The binding of BDNF to its receptor leads to the activation of several signaling pathways, including protein kinase A (PKA), mitogen-activated protein kinase (MAPK), and CREB [37]. In accordance, it has been demonstrated that swimming training protocol increased the signaling pathways of CREB and protein kinase B (AKT) activity in the hippocampus of older rats [38]. Therefore, the combined training in water should also be effective in activating the above-mentioned mechanisms.

Moreover, our study showed that the combined training in water significantly increased the hippocampal gene expression of NGF in elderly rats. Although several studies have confirmed the involvement of NGF in learning processes, its role in brain plasticity after exercise training is poorly understood. In this regard, treadmill training in rats for eight weeks has been shown to increase NGF expression and neural survival of the hippocampal gyrus [14]. In another study, moderately-intense forced treadmill training inhibited apoptosis by increasing NGF levels and activating the PI3K signaling pathway in the hippocampus of elderly rats [12]. Contrary to the results of the current study, eight weeks of swimming training (ranging from 5 min in the first week to 60 min in the last week, three sessions per week) had no significant effect on hippocampal NGF and BDNF in mice with Alzheimer's disease [13]. Considering that the exercise protocol of our study was different from that of the above study in both the intensity and the duration (combination of aerobic and strength training in water), different intensities and durations of training protocols may be one of the reasons for the lack of significant changes among our study and the above study.

Exercise training increases hippocampal plasticity in aging through several possible mechanisms, including increased neurotrophic factors such as BDNF (one of the important mediators of crosstalk between contracted skeletal muscles and the brain during exercise training, crucial for neurogenesis and synaptic plasticity) [30], enhanced synaptic plasticity (through activation the cAMP/PKA/CREB signaling pathway) [39], reduced hippocampal atrophy [40], and decreased inflammation and oxidative stress [41]. These findings underscore the importance of physical activity as a non-pharmacological intervention to maintain cognitive health and to prevent age-related decline in brain function.

### 4.2. Working memory

Aging causes changes in blood vessels in the brain. Due to the narrowing of the arteries and the diminished formation of new capillaries, blood flow to the brain can decrease [42]. Evidence shows that physical activity can improve mental and cognitive performance and plays a role in preventing the decline of cognitive performance [43]. Exercise can indirectly affect the gene expression of neurotrophic factors by affecting the secretion of neurotransmitters such as acetylcholine, gamma amino butyric acid, and monoamines [44]. There is a consensus in the literature that physical training has a positive effect on improving memory function; neurotrophins can play a very important role in this scenario, such as neurogenesis, neuronal survival, and synaptic plasticity [45].

Despite the improvements in NGF and CREB gene expression, the findings of the present study showed that the combined water training had an indirect effect on working memory in elderly rats. Some studies have demonstrated that working memory is improved by chronic exercise in older adults [30],[46],[47]. In one investigation, eight weeks of moderately-intense swimming training (30 minutes a day, 5 days a week) improved memory [20]. In another study, 4 weeks of swimming training (6 days a week, 60 min) improved spatial memory in aging rats [18]. The results of the Y-maze test after performing high-intensity exercises on the treadmill showed that there was a memory disorder in the mice [48]. In addition, three studies within a review of human samples showed that aerobic exercises had no significant effect on improving memory [49]–[51]. They reported that among the reasons for the lack of memory improvements may be variations in the training characteristics (such as frequency, intensity, duration, type, and duration of intervention) that could involve mechanisms underlying improving working memory in different ways. Another possible reason for the inconsistent results is the use of different tools and measurement tests, which made it difficult to comprehensively investigate the effect of physical training interventions on working memory using a single instrument [52]. However, considering that the rats in the present study did not have memory disorders, this factor can be considered as one of the mechanisms of the lack of significance of training in water on memory. In addition, environmental influences such as physical training on memory usually take more time to become institutionalized than genetic or hormonal changes that are affected faster than exercise training. One of the limitations of the present study was due to the multi-sectoral nature of working memory and different measurement tools to examine its different dimensions [52] and to examine memory. In addition, one of the primary limitations of this research is the absence of multiple functional tests, particularly those that also evaluate long-term memory. Additional limitaitons of this research include the lack of a young control group and the use of endurance and resistance training groups alone, which make it difficult to accurately discuss the reasons for the observed changes. Further research is required to clarify this.

## 5. Conclusion

Our findings suggested that the combined physical training in water and similar protocols had positive effects on gene factors such as NGF and CREB. The conclusions drawn from our study emphasized the importance of further investigating the specific effects of each type of physical activity. This in-depth exploration will serve as a crucial foundation to design more effective and personalized interventions, as well as for the development of health prevention and promotion policies. However, further studies are necessary to delve into the potential of this type of training on memory in order to assess a possible positive impact in preventing the risk factors associated with cognitive decline.
